# Efficiency of two protocols for maxillary molar intrusion with mini-implants

**DOI:** 10.1590/2177-6709.21.3.056-066.oar

**Published:** 2016

**Authors:** Juliana Volpato Curi Paccini, Flávio Augusto Cotrim-Ferreira, Flávio Vellini Ferreira, Karina Maria Salvatore de Freitas, Rodrigo Hermont Cançado, Fabrício Pinelli Valarelli

**Affiliations:** 1 MSc, Universidade Cidade de São Paulo (UNICID), Department of Orthodontics, São Paulo, São Paulo, Brazil.; 2 Professor, Universidade Cidade de São Paulo (UNICID), Department of Orthodontics, São Paulo, São Paulo, Brazil.; 3 Professor, Faculdade Ingá, Department of Orthodontics, Maringá, Paraná, Brazil.

**Keywords:** Corrective Orthodontics, Tooth intrusion, Bone screws

## Abstract

**Objective:**

The aim of this study was to compare the efficiency of two protocols for maxillary molar intrusion with two or three mini-implants.

**Methods:**

Twenty five maxillary first molars extruded for loss of their antagonists in adult subjects were selected. The sample was divided into two groups, according to the intrusion protocol with two or three mini-implants. Group 1 consisted of 15 molars that were intruded by two mini-implants. Group 2 consisted of 10 molars intruded by three mini-implants. Changes with treatment were analyzed in lateral cephalograms at the beginning and at the end of intrusion of maxillary molars.

**Results:**

Results showed that there was no difference in efficiency for the two intrusion protocols. It was concluded that extruded maxillary molars can be intruded with two or three mini-implants with similar efficiency.

## RESUMO

**Objetivo:**

o objetivo do presente estudo foi comparar a eficiência de dois protocolos de intrusão de molares superiores com dois e três mini-implantes.

**Métodos:**

foram selecionados 25 primeiros molares superiores extruídos por perda de seus antagonistas, de pacientes adultos. A amostra foi dividida em dois grupos, de acordo com o protocolo de intrusão, com dois ou três mini-implantes: o Grupo 1 consistiu de 15 molares que sofreram intrusão com dois mini-implantes; o Grupo 2 foi constituído por 10 molares intruídos por três mini-implantes. As alterações com o tratamento foram analisadas em telerradiografias em norma lateral ao início e ao final da intrusão dos molares superiores.

**Resultados:**

os resultados mostraram que não houve diferença na eficiência dos dois protocolos de intrusão. Concluiu-se que molares superiores extruídos podem ser intruídos, com eficiência semelhante, por meio de dois ou três mini-implantes.

## INTRODUCTION AND STATEMENT OF THE PROBLEM

One of the most difficult movements in orthodontic mechanics requiring efficient anchorage to achieve success is tooth intrusion. This movement is usually necessary when a tooth has extruded, especially due to absence of the antagonist tooth. Extrusion can cause several problems, such as occlusal interferences and consequent functional problems.^1^ ^-^ ^4^ It is, therefore, necessary to correct this condition to further promote prosthetic rehabilitation of the antagonist tooth.

There are several intra- and extraoral areas to be used as anchorage. Conventional methods present some inconvenience, including esthetic implications, anchorage loss and the need for patient's compliance, greatly compromising the success of intrusion mechanics.^2^ ^,^ ^5^ ^,^ ^6^ It is extremely necessary to differentiate the intrusion of maxillary first molars from the extrusion of adjacent teeth, which can occur when proper anchorage is not used, representing a relative intrusion and not a true one.^3^ ^,^ ^6^

The use of miniscrews and the possibility to obtain absolute anchorage has provided new perspectives for Orthodontics. It created a stable point within the oral cavity, so that movements are performed in a more controlled and predictable way, with minimal need for patient's compliance.^3^ ^,^ ^4^ Currently, there are mini-implants available in a wide variety of sizes, allowing their insertion in several locations of the maxilla and mandible.^7^ Mini-implants remained in the dental market due to several advantages, such as the absence of complex surgical procedures, low cost and great patient acceptance.^8^

Currently, intrusive mechanics of maxillary molars anchored in mini-implants uses several protocols.^3^ ^,^ ^9^ ^-^ ^12^ However, there is a concern regarding the best protocol to perform molar intrusion with maximum efficiency and the ideal number of mini-implants to be used during this mechanics.

The aim of this study was to compare the dental and skeletal changes produced by intrusion of maxillary first molars anchored in mini-implants, using two different protocols, and to evaluate the efficiency of these protocols based on the ratio between the amount and duration of intrusion.

## MATERIAL AND METHODS

This study was approved by the Ethics Research Committee of Universidade Cidade de São Paulo (UNICID) (protocol 13599774).

Sample size calculation was based on an alpha significance level of 5% (0.05) and a beta of 20% (0.20) to achieve 80% power test to detect a mean difference of 0.78 mm with standard deviation of 0.6 for maxillary molar intrusion.^24^ Thus, sample size calculation revealed the need for 10 individuals in each group.

This study was retrospective, and sample selection followed the following criteria: presence of at least an extruded maxillary first molar due to loss of the antagonist tooth, patients with no growth potential, absence of chronic systemic problems, presence of lateral cephalograms from the beginning of orthodontic treatment and from the end of intrusion, presence of completed files with information concerning the procedure for intrusion of maxillary first molars and absence of endodontic treatment in the intruded molar. None of the individuals in the sample had previous orthodontic treatment or periodontal disease in the beginning of treatment. 

According to these criteria for selecting the sample, 19 patients (four males, 15 females) were selected, 13 with unilateral and six with bilateral extrusion, thereby totalling 25 first molars which had undergone mechanical intrusion, anchored in mini-implants and associated with fixed appliances. All patients were treated by graduate students supervised by the same professor at FACSETE, Porto Velho, Rondônia, Brazil. Thus, the sample was divided into two groups, according to the protocol of two or three mini-implants used for molar intrusion.

**Figure 1:**
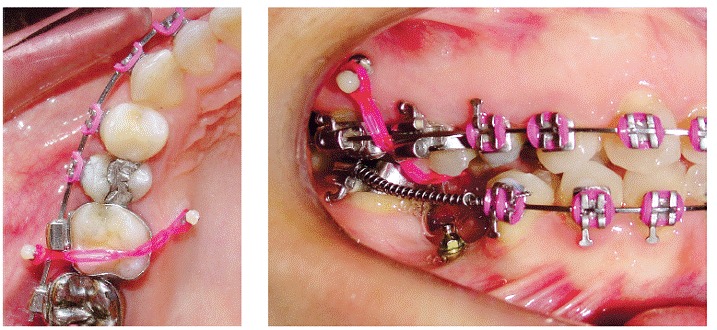
First molar intrusion in Group 1.

" Group 1 (G1): Composed of 15 maxillary first molars which were intruded by two mini-implants, one on the buccal side and one on the palatal side (Fig 1).

**Figure 2:**
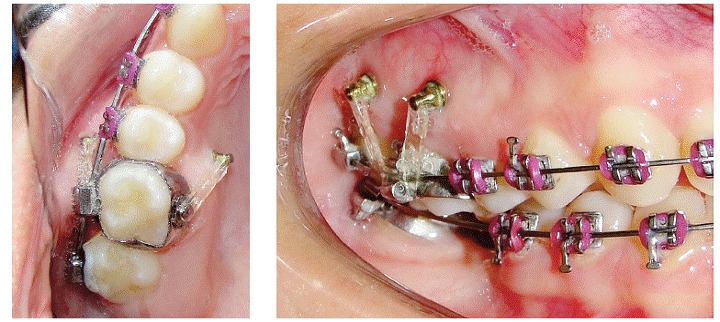
First molar intrusion in Group 2.

" Group 2 (G2): Composed of 10 maxillary first molars which were intruded by three mini-implants, two on the buccal side and one on the palatal side (Fig 2).

In patients of G1, elastomeric chains (Dental Morelli Ltda, Sorocaba, São Paulo, Brazil) were anchored in the mini-implants, passing through the occlusal surface of first molar crown (Fig 1). In patients of G2, elastomeric chains (Dental Morelli Ltda, Sorocaba, São Paulo, Brazil) were placed as follows: from the two mini-implants placed buccally to the tube of the first molar band, and from the mini-implant placed palatally to the button soldered on the first molar band, on the palatal side (Fig 2). Intrusion mechanics was applied immediately after mini-implant placement, with approximately 150 g of force being applied to each mini-implant.^13,14,15^ This force was measured by a tensiometer (50-500 g, Dental Morelli Ltda, Sorocaba, São Paulo, Brazil). The elastomeric chains were changed every four weeks and intrusion force was checked at each appointment. Retention of the intruded molars was performed with ligature wires (0.010-in).

Simultaneously to intrusion of maxillary first molars, the cases were treated with preadjusted appliances (Roth prescription, slot 0.022 x 0.028-in, Dental Morelli Ltda. Sorocaba, SP, Brazil). Patients received self-drilling mini-implants (S.I.N. Implant System, São Paulo, São Paulo, Brazil), with dimensions of 1.4 x 6 x 1 mm for the buccally installed and 1.4 x 8 x 3 mm for the palatally installed mini-implants.^16^

The mean initial age of patients was 34.25 years (SD = 8.22, minimum 22.66, maximum 46.99) for Group 1 and 39.47 years (SD = 8.12, minimum 21.07, maximum 47.44) for Group 2. Mean intrusion duration was 0.81 years (SD = 0.35, minimum 0.41, maximum 1.64) for Group 1 and 1.17 years (SD = 0.48, minimum 0.75, maximum 2.14) for Group 2.

## METHODS

Initial and final lateral cephalograms were not taken by the same equipment. Therefore, in order to increase reliability of results, correction of the magnification factor of each cephalogram was performed.^17^

Cephalograms were scanned in Microtek ScanMaker i800 (9600 x 4800 dpi, Microtek International, Inc., Carson, CA, USA) connected to a microcomputer Compaq Pavilion B6000BR board Intel Dual Core E5300 2.6 GHz, 2 GB memory RAM. Images were transferred to Dolphin Imaging Premium 5.10 software (Dolphin Imaging &Management Solutions, Chatsworth, CA, USA), through which points were marked by the same examiner and measurements were processed. The examiner was blinded regarding the group of each patient.

For better identification of maxillary first molars in the lateral cephalograms, clinical and cephalometric characteristics were associated: presence of restorations, level of extrusion, crown angulation and general characteristics of maxillary first molars as well as adjacent and antagonist teeth. Patients who had bilateral extrusions were measured twice separately.

**Figure 3:**
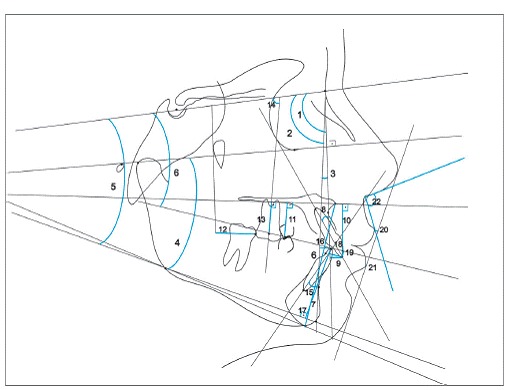
Cephalometric variables: 1) SNA, 2) SNB, 3) ANB, 4) FMA, 5) SN.GoGn, 6) SN.Ocl, 7) LAFH, 8) U1.NA, 9) U1-NA, 10) U1-PP, 11) U5-PP, 12) U6-PTV, 13) U6-PP, 14) U6.SN, 15) L1.NB, 16) L1-NB, 17) L1-GoGn, 18) Overjet, 19) Overbite, 20) UL-E, 21) LL-E, 22) Nasolabial Angle.

**Figure 4:**
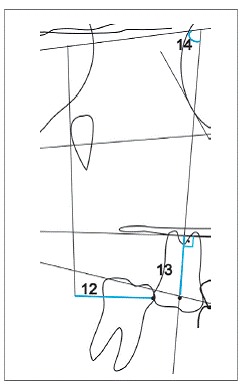
Cephalometric variables relative to the maxillary first molar: 12) U6-PTV, 13) U6-PP, 14) U6.SN.

Skeletal, dental and soft tissue variables were used, as shown in Figure 3. In initial and final cephalograms, the centroid point was built in the crown of the intruded first molar, and a vertical line was drawn perpendicular to the palatal plane, touching the centroid point. This way, the amount of intrusion of the maxillary first molar was measured. The centroid point is less influenced by potential side effects because it is a point on the longitudinal axis. Moreover, the palatal plane was used as a reference to measure intrusion of maxillary teeth^6^ (Fig 4).

To evaluate the efficiency of the two studied intrusion protocols, the following formula was used:


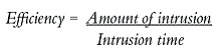


With this formula, an efficiency value for molar intrusion was determined for each group separately.

### Statistical analysis

To evaluate intraexaminer error, 15 randomly selected radiographs were remeasured after a month interval. Dependent t-test was applied to estimate systematic error. For evaluation of the random error, Dahlberg's formula was used. 

In order to check for comparability between Groups 1 and 2 regarding the initial age, independent t-test was applied. Fisher exact test was used to evaluate intergroup comparability in relation to sex and type of malocclusion at the beginning of the study.

Independent t-test was used to compare variables between Groups 1 and 2 at the initial stage and during the intrusion period. The independent t-test was also used to compare intrusion duration between groups as well as intrusion efficiency. All statistical analyses were performed with Statistica for Windows software (Statsoft, Tulsa, Oklahoma, USA). Results were considered significant for *p* < 0.05.

## RESULTS

No systematic error was detected and random errors varied from 0.18 mm (UL-E) to 0.47 mm (U6-PTV) in linear measurements and from 0.21° (FMA) to 0.95° (ANB). The groups were compatible regarding age, sex and type of malocclusion (Tables 1, ^2^ and ^3^). Table 4 showed that groups were also cephalometrically compatible at the beginning of treatment. During treatment/intrusion phase, only the variable LL-E showed statistically significant difference between groups (Table 5).

**Table 1:** Intergroup comparability of initial age (independent t-test).

Variable (Years)	Group 1		Group 2		
	Mean	SD	Mean	SD	P Value
Initial age	34.25	8.22	39.47	8.12	0.131

**Table 2:** Intergroup comparability of sex distribution (Fisher exact test).

Sex / Group	Female	Male	Total
Group 1	12	3	15
Group 2	8	2	10
Total	20	5	25
Fisher Exact Test		DF=1	*p* = 1.000

**Table 3:** Intergroup comparability of type of malocclusion (Fisher exact test).

Type of malocclusion	Group 1 (n = 15)	Group 2 (n = 10)
Class I	8	3
Class II	7	7
Fisher Exact Test	DF=1	*p* = 0.413

**Table 4:** Intergroup comparison of cephalometric variables at the initial stage (T_1_) (independent t-tests).

Variables	Group 1		Group 2		*p* value
	Mean	SD	Mean	SD	
Maxillary Component					
SNA (degrees)	85.18	3.20	85.56	3.81	0.7897
Mandibular Component					
SNB (degrees)	81.43	3.75	81.48	2.20	0.9681
Maxillomandibular Relationship					
ANB (degrees)	3.77	2.41	4.08	4.75	0.8328
Vertical Component					
FMA (degrees)	27.51	4.96	27.76	6.03	0.9119
SN.GoGn (degrees)	29.98	5.63	30.60	4.55	0.7744
SN.Ocl (degrees)	6.08	7.05	7.96	3.67	0.4477
LAFH (mm)	63.24	5.32	60.12	6.98	0.2174
Maxillary Dentoalveolar Component					
U1.NA (degrees)	26.01	8.00	23.61	8.19	0.4747
U1-NA (mm)	4.80	2.77	3.15	2.76	0.1572
U1-PP (mm)	26.98	3.26	24.88	3.06	0.1196
U5-PP (mm)	23.82	2.68	22.11	4.10	0.2177
U6-PTV (mm)	19.65	2.75	19.47	3.53	0.8853
U6-PP (mm)	21.58	2.83	19.79	3.17	0.1530
U6.SN (degrees)	80.81	5.30	81.00	6.92	0.9376
Mandibular Dentoalveolar Component					
L1.NB (degrees)	27.33	5.94	22.34	5.94	0.0514
L1-NB (mm)	5.83	2.22	3.98	2.26	0.0548
L1-GoGn (mm)	37.45	2.47	37.11	3.94	0.7944
Dental Relationships					
Overjet (mm)	3.84	1.11	4.32	2.71	0.5437
Overbite(mm)	2.99	0.94	3.49	2.38	0.4717
Soft Tissue Component					
UL-E (mm)	-3.75	2.43	-4.24	3.84	0.6965
LL-E (mm)	-1.21	2.40	-1.65	2.96	0.6835
Nasolabial angle (degrees)	100.56	7.45	103.48	11.60	0.4495

**Table 5:** Intergroup comparison of cephalometric changes during treatment/intrusion (T_2_-T_1_) (independent t -ests).

Variables	Group 1		Group 2		*p* value
	Mean	SD	Mean	SD	
Maxillary Component					
SNA (degrees)	0.06	1.42	0.36	1.43	0.6104
Mandibular Component					
SNB (degrees)	-0.09	1.27	0.48	0.90	0.2295
Maxillomandibular Relationship					
ANB (degrees)	0.13	0.99	-0.11	1.33	0.6134
Vertical Component					
FMA (degrees)	-0.47	1.60	-1.24	0.98	0.1863
SN.GoGn (degrees)	0.34	1.27	-0.57	1.20	0.0864
SN.Ocl (degrees)	4.81	3.66	3.44	3.39	0.3539
LAFH (mm)	-0.16	2.11	0.16	1.80	0.6978
Maxillary Dentoalveolar Component					
U1.NA (degrees)	2.33	4.98	2.13	7.76	0.9368
U1-NA (mm)	0.52	1.88	-0.12	1.97	0.4215
U1-PP (mm)	-0.59	3.23	1.73	9.32	0.3808
U5-PP (mm)	-1.39	1.90	-1.31	1.67	0.9183
U6-PTV (mm)	-0.08	2.66	0.50	2.62	0.5963
U6-PP (mm)	-1.79	1.28	-2.12	1.25	0.5253
U6.SN (degrees)	1.17	3.29	-0.42	5.02	0.3458
Mandibular Dentoalveolar Component					
L1.NB (degrees)	2.51	2.80	0.21	4.07	0.1059
L1-NB (mm)	0.33	0.95	0.15	1.13	0.6761
L1-GoGn (mm)	-0.75	1.18	-0.37	1.42	0.4780
Dental Relationships					
Overjet (mm)	0.55	1.32	-0.51	2.23	0.1484
Overbite(mm)	-1.49	1.24	-1.57	2.46	0.9117
Soft Tissue Component					
UL-E (mm)	0.30	1.98	-0.13	2.24	0.6188
LL-E (mm)	1.11	1.02	-0.65	2.65	0.0275*
Nasolabial angle (degrees)	-1.07	8.92	-4.44	8.73	0.3601

* Statistically significant for *p* < 0.05

There was statistically significant difference for the time of intrusion, but there was no significant difference regarding the efficiency of intrusion between the two groups (Table 6).

**Table 6:** Intergroup comparison of intrusion duration and efficiency (independent t-tests).

Variables	Group 1		Group 2		*P* Value
	Mean	SD	Mean	SD	
Intrusion duration (years)	0.81	0.35	1.17	0.48	0.045*
Intrusion efficiency	-2.18	1.14	-1.86	1.07	0.489

* Statistically significant for *p* < 0.05.

## DISCUSSION

An important criterion for sample selection was to include only patients with no growth potential. In a growing patient, vertical maxillary growth and development could possibly result in a relative molar intrusion, i.e., it would be questionable whether an actual intrusion occurred or presented as a result of alveolar process growth.^6^

Patients with systemic diseases, such as diabetes, osteoporosis, heart disease, clotting disorders and metabolic bone disorders, were excluded from the sample, as these factors could influence root resorption and stability of mini-implant and consequently in treatment/intrusion time.^18^ Endodonticaly treated teeth were also excluded from the sample, since they could present an injury in healing process or root resorption, and these factors could influence the amount of intrusion.^18^ ^,^ ^19^

The study sample consisted of two lateral cephalograms of each patient. Lateral cephalograms for evaluation of skeletal and dental changes produced by intrusion mechanics are widely used in the literature, including assessment of maxillary molar intrusion.^4^ ^,^ ^20^ ^,^ ^21^ Dolphin Imaging software computerized method also minimized errors in the determination of cephalometric values.^22^ Several authors have used this software in other studies, thus ensuring its reliability.^22^

Groups were compatible regarding initial age (Table 1), sex distribution (Table 2), type of malocclusion (Table 3) and cephalometry at the beginning of treatment (Table 4). This allows comparability of groups, excluding factors influencing the results.

The sample was retrospectively selected, and there was probably some influence of the amount of intrusion required regarding the choice of protocols with two or three mini-implants. This possibly generated a difference between groups regarding the amount of intrusion achieved, being higher in the group in which the three mini-implant protocol was used (Table 5). This fact also explains the longer intrusion duration of this group (Table 6). However, to minimize this difference, intrusion efficiency was compared, which is the amount of intrusion achieved divided by intrusion duration, thus allowing intergroup comparison.

### Cephalometric changes

During treatment/intrusion phase, there was no difference in skeletal and dental changes, except for the variable LL-E that showed a statistically significant difference between groups (Table 5).

**Figure 5:**
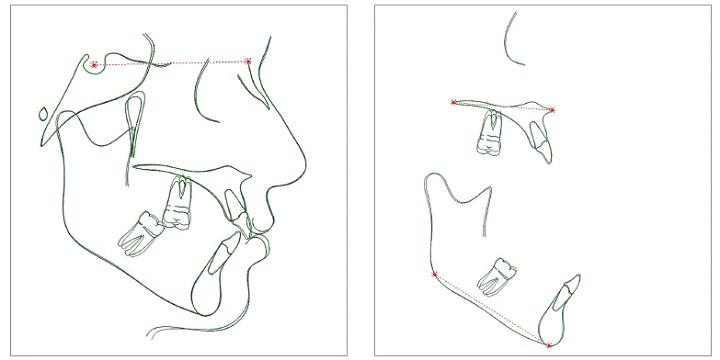
Initial and final average tracings superimposition of Group

According to specific first molar variables, i.e. U6-PTV, U6-PP and U6.SN, it was observed that both G1 and G2 showed a significant reduction in U6-PP during treatment, demonstrating effectiveness of the intrusion mechanics. In G1, mean intrusion of the maxillary first molar of 1.79 mm was obtained; while for G2, the mean intrusion of the first molar was of 2.12 mm. Mean molar intrusion was similar between groups (Table 5, Figs 5 and ^6^). Molar intrusion was finished when the tooth was leveled with adjacent teeth. Therefore, the amount of intrusion ranged from 0.6 to 5 mm, which was reasonable considering the different amount of overeruption of the tooth in each patient. The amount of intrusion varied in the literature according to the clinical needs. Carrillo et al^23^ achieved 1.2 to 2.3 mm, Heravi et al^24^ ranged from 1.5 to 4.5 mm and Al-Fraidiand Zawawi^25^achieved 4 mm in their studies. 

**Figure 6:**
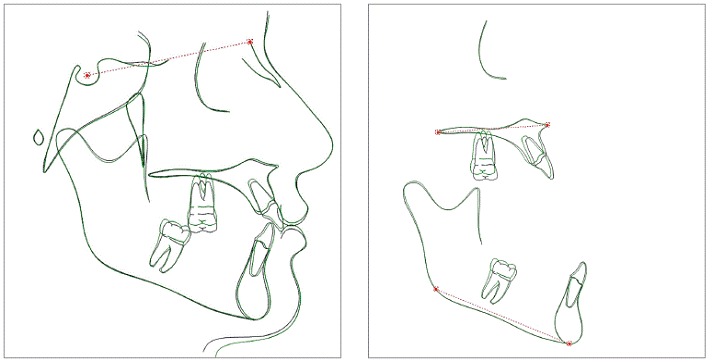
Initial and final average tracings superimposition of Group 2.

There was also intrusion of second maxillary premolars in both groups (mean of 1.39 and 1.31 mm for Groups 1 and 2, respectively); however, without significant difference between them (Table 5). Intrusion of premolars and molars was caused by intrusion mechanics with mini-implant anchorage. Since a leveling arch was used in fixed appliances in maxillary premolars and molars, this result was already expected. If the orthodontic mechanics of leveling and alignment was being held without intrusive force in the maxillary first molar region, premolars would probably extrude.^26^ In the work by Yao et al,^3^ there was a mean intrusion of first molars and second premolars of 3 mm and 2 mm, respectively.^3^ These results corroborate the present study, since they show that the intrusion mechanics of the first molar also provides intrusion of the second premolar.

Both protocols in this study used forces from buccally and palatally placed mini-implants to prevent the overerupted molar from tipping either labio-palatally or mesio-distally as it was intruded. There was a small variation, in both groups, in mesiodistal angulation and anteroposterior movement of maxillary molars (U6.SN and U6-PTV, respectively, Table 5). This evinced a purely intrusive mechanics, without molar angulation that could camouflage the vertical positioning of these teeth.^27^

The method used in this study for molar intrusion produced an excellent control of labio-palatal maxillary molar position during intrusion with elastomeric chains attached to the mini-implants.

There is no agreement in the literature on the optimum force to be used for molar intrusion. Some authors suggest forces ranging from 30 to 100 g,^24^ ^,^ ^28^ whereas others have recommended using greater force for intrusion (150 to 500 g).^29^ ^,^ ^30^ In this study, approximately 150 g of force was delivered from a short length of elastomeric chain. Force was carefully measured to ensure that it did not exceed the desired force level.

Regarding intrusion duration, there was statistically significant difference between groups, indicating that Group 2, the protocol with three mini-implants, showed longer intrusion duration, when compared to Group 1, the protocol with two mini-implants. However, these results are influenced by the greater or lesser need for intrusion in each case, as described above. 

Maybe it is interesting that the tooth with the greatest need for intrusion has three mini-implants placed, so as to increase reinforcement of anchorage.

There was no significant difference regarding intrusion efficiency between the two groups (Table 6).

## CONCLUSION

Protocols of maxillary molar intrusion with two or three mini-implants presented the same efficiency of skeletal anchorage.
